# Supplementary material for: Elongator Subunit 3 (Elp3) Is Required for Zebrafish Trunk Development
Source: Int J Mol Sci. 2020 Jan 31;21(3):925. doi: 10.3390/ijms21030925 (PMC7036906; doi:10.3390/ijms21030925)
Supplement: Supplementary file 1 [file ijms-21-00925-s001.pdf]

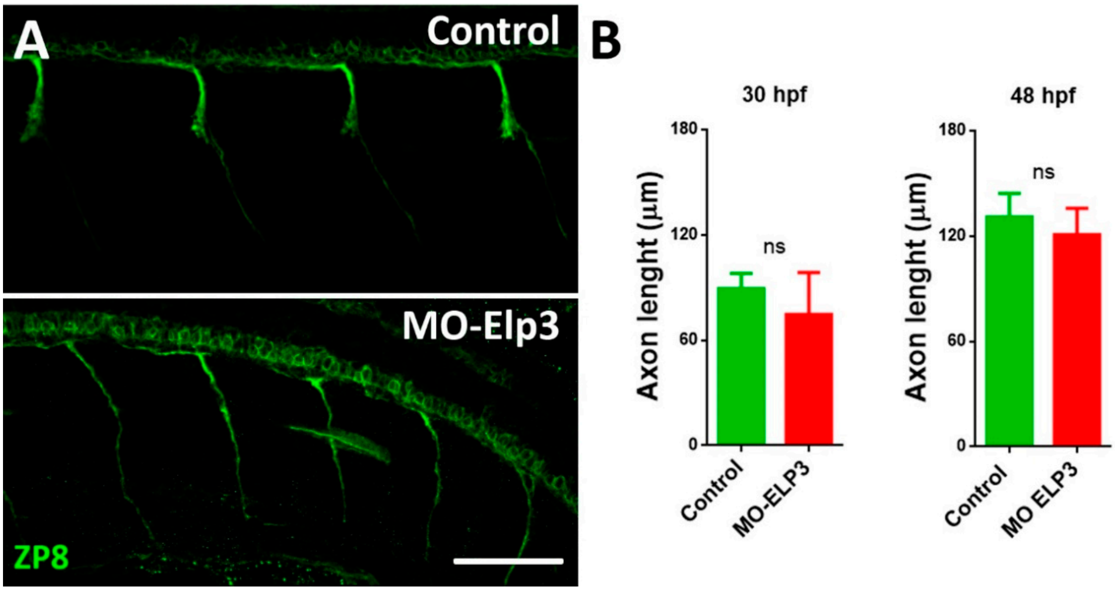

Supplementary Figure S1. Motor neuron axon length is not affected by Elp3 knock down. (**A**) Representative confocal images of motor neuron, stained with ZN8 (CD166 antigen homolog A, Neurolin) 48 hpf of control and morphant embryos (Bar 20 μm). (**B**) Quantification of axonal length at 30 and 48 hpf of control and morphant embryos ($N = 3$, $n = 10$, per embryo).
